# An evaluation of rehabilitation students’ learning goals in their first year: a text mining approach

**DOI:** 10.3389/fmed.2024.1239916

**Published:** 2024-03-13

**Authors:** Shin Kitamura, Kotaro Takeda, Shintaro Uehara, Taiki Yoshida, Hirofumi Ota, Shigeo Tanabe, Kazuya Takeda, Soichiro Koyama, Hiroaki Sakurai, Yoshikiyo Kanada

**Affiliations:** Faculty of Rehabilitation, School of Health Sciences, Fujita Health University, Aichi, Japan

**Keywords:** cluster analysis, professional education, rehabilitation, students, text mining

## Abstract

**Introduction:**

Qualitative information in the form of written reflection reports is vital for evaluating students’ progress in education. As a pilot study, we used text mining, which analyzes qualitative information with quantitative features, to investigate how rehabilitation students’ goals change during their first year at university.

**Methods:**

We recruited 109 first-year students (66 physical therapy and 43 occupational therapy students) enrolled in a university rehabilitation course. These students completed an open-ended questionnaire about their learning goals at the time of admission and at 6 and 12 months after admission to the university. Text mining was used to objectively interpret the descriptive text data from all three-time points to extract frequently occurring nouns at once. Then, hierarchical cluster analysis was performed to generate clusters. The number of students who mentioned at least one noun in each cluster was counted and the percentages of students in each cluster were compared for the three periods using Cochran’s Q test.

**Results:**

The 31 nouns that appeared 10 or more times in the 427 sentences were classified into three clusters: “Socializing,” “Practical Training,” and “Classroom Learning.” The percentage of students in all three clusters showed significant differences across the time periods (*p* < 0.001 for “Socializing”; *p* < 0.01 for “Practical Training” and “Classroom Learning”).

**Conclusion:**

These findings suggest that the students’ learning goals changed during their first year of education. This objective analytical method will enable researchers to examine transitional trends in students’ reflections and capture their psychological changes, making it a useful tool in educational research.

## 1 Introduction

Data analysis methods have rapidly evolved in biomedical, life, and social sciences (1). The analytical methods commonly taught in introductory statistics courses (e.g., *t*-test, analysis of variance) have been extensively used. However, their usage has been declining in recent years (1). On the other hand, the use of multivariate statistical and machine learning approaches, though less prevalent than the aforementioned analytical methods, has been expanded due to the increasing accessibility of large and intricate datasets (2). In recent years, these methods have been applied in educational science. Notably, data mining, the process of extracting patterns and relationships in data from large datasets has been applied to massive student educational datasets to interpret their academic performance (3).

Educational data mining is the application of data mining techniques to educational data, and its objective is to resolve educational research issues (3). Educational data mining is mainly applied to educational tasks such as the analysis and visualization of educational data, providing feedback for supporting instructors, recommending personalized learning contents, predicting student performance, developing a cognitive model for students that includes their skills and knowledges, detecting undesirable student behaviors, and grouping students (3). Recently, with the development of educational software, the expansion of databases of student information, and the development of web-based education such as e-learning, a large amount of information is being generated, and further utilization of educational data is expected (3). The goal of educational data mining is subjective, such as improving the learning process for students. As many different types of data in education have become publicly available in recent years, there has been a need to utilize subtle measurement techniques that can be adapted to a variety of data (4). For example, the evaluation of students’ progress and changes in their learning goals cannot only be conducted quantitatively based on numerical data interpretation expressed on an interval or ordinal scale but also through qualitative analysis that includes essential elements that evaluate students’ progress and change in their learning goals. In fact, in health professional education, descriptive reports provide reflections and students’ self-evaluations, used as a means of professional development, which provides objective information for identifying students’ progress (5–7). Text mining is a data mining method that objectively analyzes qualitative data and is being utilized in supportive care for preterm children (8), education of university students, including research on cognitive control functions of students (9) and education of health professionals (10–16). This method contributes to the objective interpretation of text data by modeling important concepts and calculating the co-occurrence of keywords that occur frequently in the text.

Most of the existing text-mining studies on learning in medicine and healthcare focused on medical students (11, 14, 15). These studies have primarily analyzed reflection reports related to learning within specific educational programs, such as clinical practice and off-campus classes, at different points in time. A previous study examining the relationship between students’ learning goals and their academic performance demonstrated that text-mining techniques can be employed to identify their goals objectively and systematically, proving valuable in enhancing the understanding of diverse student needs (17). In education, setting learning goals play a crucial role in academic performance (18, 19). It was emphasized that it is important [1] for students to set their own learning goals to foster the awareness of their strengths and weaknesses, and [2] for educators to understand that these goals will change as students learn (20). It has also been demonstrated that, in order to enhance the educational impact of setting learning goals, educational supports are essential in terms of both utilizing these goals and receiving feedback from teachers (21, 22). On the other hand, for a comprehensive assessment of students’ development, it is crucial not only to comprehend their learning goals at a particular juncture in the entire educational program but also to track their transitions. However, no study has examined changes in students’ learning goals over time using text mining. In summary, there is a scarcity of studies on text mining that specifically target physical therapy students (PTSs) or occupational therapy students (OTSs) and the effects of their long-term learning.

In the present pilot study, we proposed a method that utilizes text mining to track the evolution of students’ learning goals in health professional education. We collected descriptive text data at regular intervals from both PTSs and OTSs. By applying text mining to the collected text data across various time periods, our objective was to gain insights into the changes that took place in their learning goals throughout their educational journey.

## 2 Materials and methods

The present study retrospectively analyzed longitudinal descriptive text data on the learning goals of students in a university rehabilitation course in Japan. The analysis in this study was conducted on text data written in Japanese.

We recruited 109 first-year students who enrolled in the course of PT and OT at the Faculty of Rehabilitation, School of Health Sciences, Fujita Health University, in April 2021. This number includes all first-year students, except those who were repeating the course. Table 1 displays the distribution of students by major and gender. Although PT and OT are different healthcare professions, students receive similar health professional education in the first year at this university, including physiology, anatomy, bioethics, social work, statistics, and physics. Therefore, we did not distinguish between PTS and OTS in the present study. This study was approved by the Ethics Review Committee of Fujita Health University (approval number HM21-377) and conducted in accordance with the Declaration of Helsinki.

**Table 1:** Majors and gender of the participating students.

Majors	Male	Female	Total
PTS, *n*	32	34	66
OTS, *n*	13	30	43
Total, *n*	45	64	109

PTS, physical therapy students; OTS, occupational therapy students.

The students answered the following open-ended questions at three different time points; that is, at the time of admission (at 0 months), 6 months (after completing the first semester), and 12 months (after completing the first year) on entering school:

Q_0mo_: What do you want to work on first in school?

Q_6mo_: What do you want to work on in the second semester of your first year?

Q_12mo_: What do you want to work on in your second year?

These questionaries were provided using a learning result visualization system in which students periodically self-evaluated their goals and achievements (23). The purpose and content of the questionnaire and the times when the students would be asked to respond were explained to the students prior to the survey. The students responded by typing the text with no time limit, using their own computer tablets on an assigned day. The response time was approximately 20 min.

To globally capture the student goals from the descriptive words and their changes throughout the first year of education, text mining and hierarchical cluster analysis (HCA) were performed using a software KH Coder (24, 25) on the text data of the questionnaire responses. As for preparation, one of the authors (SKi) manually corrected typographical errors in each sentence. Text data from all the time periods (Q_0mo_, Q_6mo_, and Q_12mo_) were pooled together. Nouns were detected as a first step in text mining. If synonyms were identified, they were unified into a single word after ensuring that the meaning of the sentence remained unchanged. “Physical therapy” and “occupational therapy” were treated as one word. Words that indicated the timing of the responses, such as “first semester” and “second semester,” were excluded. Only nouns whose frequency of appearance was more than 10 across all three time periods were selected for the HCA. Ward’s method, which minimizes the total within-cluster variance and maximizes the between-cluster variance, was employed in HCA (26). We classified the nouns into clusters using the agglomeration dissimilarity coefficient based on the Jaccard distance as a measure of cooccurrence for term pairs, and the resultant HCA dendrogram was located by KH Coder (27). The authors (SKi, KoT, SU, TY, HO, and ST) discussed the determination of the threshold of the agglomeration dissimilarity coefficient for easy interpretation and naming of each cluster.

To examine how student goals changed, the number of students who wrote sentences containing at least one noun constituting each cluster was counted for each time period. This helped to examine the change in students’ goals within clusters across time periods. Cochran’s Q test was used to compare the proportions of students over time in each cluster. McNemar’s test was used to compare the three periods within each cluster, with a statistical significance level of 0.05/3 = 0.017 according to the Bonferroni correction. Statistical Package for Social Sciences (SPSS; Version 28, IBM Corp., Armonk, NY, United States) was used for statistical analysis.

## 3 Results

**Figure 1:**
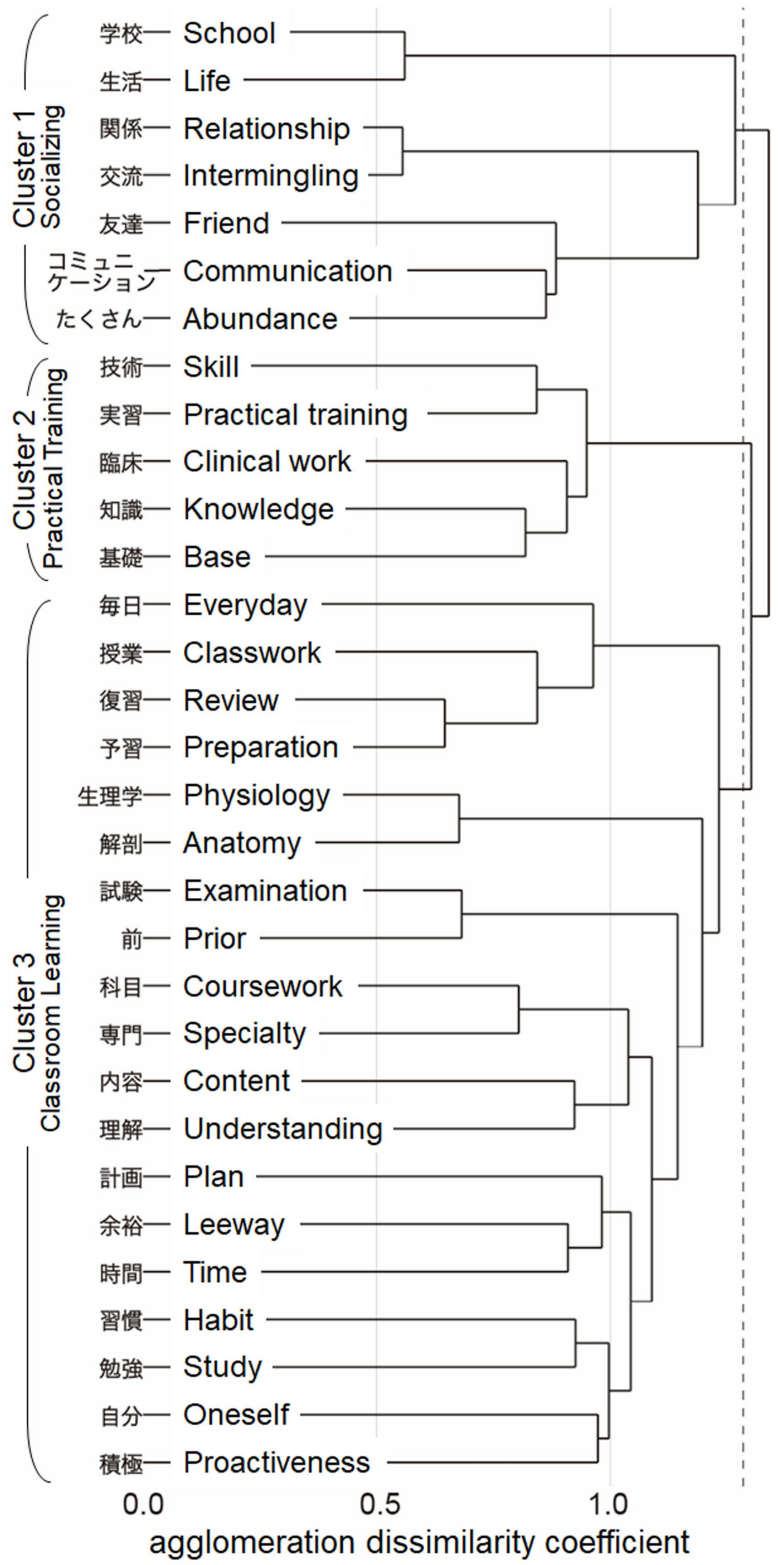
Cluster Dendrogram Nouns extracted from the text data of students’ responses across all time periods were classified into three clusters: Socializing, Practiced Training, and Classroom Learning. English words corresponding to each Japanese noun are also shown for display purposes. Note that a few Japanese words cannot be expressed with a single English word, but require two words. The dotted vertical line represents the threshold of the agglomeration dissimilarity coefficient (approximately 1.25) that determines the number of clusters.

Of the 427 sentences answered by the 109 students, 7,034 words consisting of 781 morphemes were identified. Among these words, 281 nouns, with a total of 1,389 occurrences, were extracted (Table 2). We classified the 31 nouns that appeared more than 10 times into three clusters using HCA. The clusters were named “Socializing,” “Practical Training,” and “Classroom Learning” (Figure 1).

**Table 2:** Basic statistics of text mining.

Category	Metrics
Number of sentences	427
Number of morphemes type	781
Average number of morphemes per response	22
Number of nouns type	281
Number of nouns	1,389
Average number of nouns per response	4

**Figure 2:**
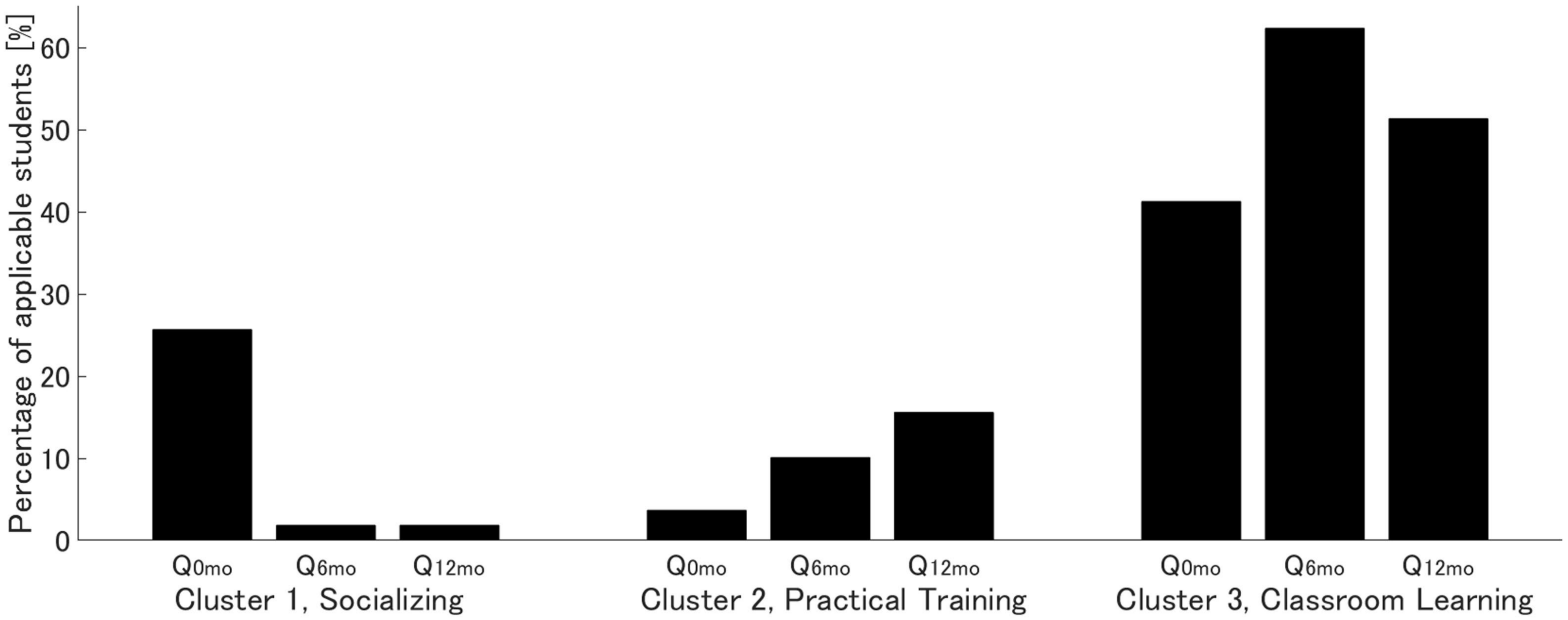
Changes in the ratio of applicable students in each cluster The bar graphs represent the percentage of students who responded with words comprising each cluster at each time point (Q_0mo_, at admission; Q_6mo_, 6 months later; Q_12mo_, 12 months later). Note that the sum of the percentages of the three clusters in each period does not add up to 100% because it includes students belonging to more than one cluster or none of the clusters.

Figure 2 shows the percentage of students who responded to the words in each cluster at each time period, and Table 3 shows the results of comparisons across the three time periods within clusters. The number of words for the cluster “Classroom Learning” was greater for the majority of students in comparison to other clusters at all time periods. Cochran’s Q test showed significant differences in the percentage of the number of students in the three time periods in all clusters: “Socializing,” “Practical Training,” and “Classroom Learning.” The number of students who described the words in the “Socializing” cluster was significantly higher at the time of admission than at any other time points, with less than 2% of students describing the words at Q_6mo_ and Q_12mo_. The number of students who described words in the cluster “Practical Training” gradually increased over the course of the year and was significantly higher in Q_12mo_ than at the time of admission. The highest number of students who described words for the cluster of “Classroom Learning” was significantly higher at Q_6mo_than at the time of admission.

**Table 3:** Comparison across three time periods in each cluster.

Cluster	Number of responded students, *n* (%)			Cochran’s Q test chi-square (*p* value)	McNemar’s test standardized statistic |*z*| (*p* value)		
	Q_0mo_	Q_6mo_	Q_12mo_		Q_0mo_ vs. Q_6mo_	Q_0mo_ vs. Q_12mo_	Q_6mo_ vs. Q_12mo_
Socializing	28 (25.7)	2 (1.8)	2 (1.8)	42.45 (<0.01)	5.62 (<0.01)	5.62 (<0.01)	1.00 (0.00)
Practical Training	4 (3.7)	11 (10.1)	17 (15.6)	10.16 (<0.01)	1.71 (0.08)	3.18 (<0.01)	1.47 (0.14)
Classroom Learning	45 (41.3)	68 (62.4)	56 (51.4)	10.05 (<0.01)	1.51 (0.13)	3.16 (<0.01)	1.65 (0.09)

Percentage of students represents the value relative to the total number of students (*n* = 109). *p*-values in the table are uncorrected.

## 4 Discussion

This pilot study applied text-mining methods to evaluate rehabilitation students’ progress longitudinally. We analyzed descriptive textual data from 109 PTS and OTS about their planned content of focus during their first year of university education at three different time points. Students’ responses were grouped and classified into three clusters: “Socializing,” “Practical Training,” and “Classroom Learning.” The percentage of students who responded with the corresponding words differed significantly in the three periods: before, during, and after the first year of education for the three clusters. While previous studies have shown that health professional students’ perceptions of learning and attitudes toward professionalism evolve as they progress through higher grades (28, 29), this study clearly demonstrates that students’ attitudes on learning, toward the specialization fields, change over the course of a single year by demonstrating changes in their learning goals.

The proportion of students who responded with words associated with the “Socializing” cluster was high at the time of admission and almost disappeared thereafter. At the time of admission, approximately 25% of the students expressed their desire to work on “Socializing,” indicating anxiety that the majority of them experienced about interacting with others in the school. This anxiety disappeared within 6 months of admission as new friendships were established.

The proportion of students who responded with words related to the “Practical Training” cluster gradually increased over the course of the year. The first year is crucial for students who aspire to become rehabilitation professionals because they realize the value of professional work (29). It is assumed that the students’ motivation to engage in clinical practice gradually increased with exposure to more specialized subjects throughout the year. In addition, the university’s curriculum includes a clinical internship for second-year students, which can be a source of anxiety and stress because it involves learning in an environment different from their usual school life (30–34). Thus, the motivation to learn more about “Practical Training” could be attributed to alleviating this anxiety. The students receive more opportunities to study specialized subjects, including clinical internships as they advance to higher grades. Therefore, students’ motivation to learn about “Practical Training” will continue to rise once they move on to second grade.

The proportion of students who responded with the words in “Classroom Learning” was higher than the other clusters in all the three response periods, with its peak at 6 months after their admission. This trend is probably due to the abundance of opportunities for fundamental classroom learning in the first year, with the curriculum emphasizing the acquisition of foundational knowledge. The increase in the number of students at the 6 months marks for the words under “Classroom Learning” is attributed to having taken lectures and exams for the first time at the university, which helped in identifying their individual issues and increased their motivation to improve.

Previous studies that used text mining to identify trends in health professional students’ experiences and reflections on specific courses, such as clinical internships, conducted cross-sectional analyses (14–16). While cross-sectional analyses are useful for assessing students’ perceptions at a single point in time, longitudinal analyses are required to assess changes in their perceptions over time. In a longitudinal analysis of medical students’ reflections, a study compared the occurrence of the four most frequently used words (“responsibility,” “pride,” “knowledge/skill,” and “patient”) in their written reflections before, during, and after the clinical exposure program (12). This study found significant changes in the students’ perceptions of the characteristics of professionals working in hospitals throughout the program. It is possible to identify general trends in students’ psychological changes by examining the progress in the frequency of word occurrence. However, because students may not express their thoughts using the same words, it is appropriate to assess their progress based on themes consisting of multiple words. This provides a more accurate understanding of psychological changes. If text mining is conducted in each assessment period, themes consisting of the same words may not necessarily be generated in each period and it may be difficult to compare themes across periods. Therefore, in the present study, we conducted text mining using data from all time periods (i.e., admission, 6 months, and 12 months). We identified common themes (clusters) throughout the three periods and compared the number of students who expressed at least one noun in sentences comprising each theme across the three time periods. The analytical method proposed in this study will enable a more objective examination of transitional trends in students’ reflections and can be utilized in capturing psychological changes in students engaged in university courses such as lectures and clinical internships.

## 5 Strengths and limitations

The text mining method proposed in this pilot study facilitates objective analysis by quantitatively processing qualitative data. When employing conventional qualitative analysis methods, there exists a potential bias influenced by researchers’ experiences and perspectives (35). To address this concern, analytical methods like triangulation have been utilized to ensure validity by incorporating multiple analytical viewpoints, often involving multiple researchers in data analysis. However, this approach can be resource-intensive and impractical for handling substantial data volumes (35). Text mining is a useful analytical approach when objectively analyzing data based on a large sample such as data in educational research. The findings of this text-mining study can be regarded as more objective than those derived from traditional qualitative analysis methods.

The changes in students’ learning goals identified in the present study can be used to improve learning support in educational settings. For instance, the finding that approximately 25% of the recruited students set “Socializing” as a goal at the time of admission indicates that it is advisable to provide early support after admission to increase opportunities for communication among students, such as through group work. While “Practical Training” was initially at a relatively low level, awareness gradually increased over the course of the year. However, additional efforts may be needed to further increase awareness in preparation for clinical practice, which commences in the second year. “Classroom Learning” was consistently mentioned by approximately half of the students. It is crucial to convey the importance of subjects related to fundamental learning and their connection with clinical practice to further enhance their awareness. The active involvement of teachers in these transitions in students’ attitudes will make learning more motivating and effective.

The proposed method involves extracting words that appeared frequently in the responses from all students. It also evaluates the learning goals mentioned by a substantial number of students and examines their transitional trends. In other words, this approach estimates the overall student trends. In education, personalized learning support may be needed for each student, and it is important to analyze individual goals and their changes. In the future, the proposed method can be applied to further support individual learning by improving the analytical method to quantitatively show individual student characteristics in learning goals by comparing overall trends with individual goals.

## 6 Conclusion

The present pilot study applied text-mining methods to objectively identify changes in rehabilitation students’ learning goals during their first year of education. The study demonstrates that students’ learning goals change during their first year at university. The analytical method proposed in this study enables capturing the psychological changes of students and could be a useful method in educational research.

## Data availability statement

The raw data supporting the conclusions of this article will be made available by the authors, without undue reservation.

## Ethics statement

The studies involving humans were approved by Ethics Review Committee of Fujita Health University. The studies were conducted in accordance with the local legislation and institutional requirements. The ethics committee/institutional review board waived the requirement of written informed consent for participation from the participants or the participants’ legal guardians/next of kin because Informed consent was obtained in the form of an opt-out, and an oral explanation was also given at the time of data collection.

## Author contributions

KoT, SKi, and SU contributed to the study concept and design, data analysis and interpretation, and manuscript writing. TY, HO, and ST contributed to the study concept and design, analysis, and interpretation of the data. KaT contributed to the study concept, study design, and data collection. SKo, HS, and YK contributed to the study concept, design, and data interpretation. All authors contributed to the article and approved the submitted version.

## References

[1] Bolt T Nomi JS Bzdok D Uddin LQ. Educating the future generation of researchers: a cross-disciplinary survey of trends in analysis methods. PLoS Biol. (2021) 19:e3001313. doi: 10.1371/journal.pbio.3001313, PMID: 34324488 PMC8321514

[2] Fan J Han F Liu H. Challenges of big data analysis. Natl Sci Rev. (2014) 1:293–314. doi: 10.1093/nsr/nwt032, PMID: 25419469 PMC4236847

[3] Romero C Ventura S. Educational data mining: a review of the state of the art. IEEE Trans Syst Man Cybern C. (2010) 40:601–18. doi: 10.1109/tsmcc.2010.2053532

[4] Romero C Ventura S. Educational data mining: a survey from 1995 to 2005. Expert Syst Appl. (2007) 33:135–46. doi: 10.1016/j.eswa.2006.04.005

[5] Charon R Hermann N. Commentary: a sense of story, or why teach reflective writing? Acad Med. (2012) 87:5–7. doi: 10.1097/acm.0b013e31823a59c7, PMID: 22201631 PMC3247912

[6] Sandars J. The use of reflection in medical education: AMEE guide no. 44. Med Teach. (2009) 31:685–95. doi: 10.1080/01421590903050374, PMID: 19811204

[7] Schei E Fuks A Boudreau JD. Reflection in medical education: intellectual humility, discovery, and know-how. Med Health Care Philos. (2019) 22:167–78. doi: 10.1007/s11019-018-9878-2, PMID: 30460425

[8] Park JH Lee H Cho H. Analysis of the supportive care needs of the parents of preterm children in South Korea using big data text-mining: topic modeling. Child Health Nurs Res. (2021) 27:34–42. doi: 10.4094/chnr.2021.27.1.34, PMID: 35004495 PMC8650870

[9] Kase T Kawagoe T. Life skills link to mind wandering among university students: an exploratory study. Front Psychol. (2021) 12:729898. doi: 10.3389/fpsyg.2021.729898, PMID: 34707540 PMC8542690

[10] Brscic M Contiero B Schianchi A Marogna C. Challenging suicide, burnout, and depression among veterinary practitioners and students: text mining and topics modelling analysis of the scientific literature. BMC Vet Res. (2021) 17:294–10. doi: 10.1186/s12917-021-03000-x, PMID: 34488757 PMC8419380

[11] Nakamura K Kanke S Hoshi G Toyoda Y Yoshida K Kassai R. Impact of general practice / family medicine clerkships on Japanese medical students: using text mining to analyze reflective writing. Fukushima J Med Sci. (2022) 68:19–24. doi: 10.5387/fms.2021-24, PMID: 35135909 PMC9071354

[12] Shikama Y Chiba Y Yasuda M Stanyon M Otani K. The use of text mining to detect key shifts in Japanese first-year medical student professional identity formation through early exposure to non-healthcare hospital staff. BMC Med Educ. (2021) 21:389. doi: 10.1186/s12909-021-02818-1, PMID: 34284770 PMC8293517

[13] Uejima T Ota I Hamaguchi M Shigeoka H Kurita T Hiraide A. Medical students’ perceptions of emergency medical care before and during the coronavirus disease 2019 pandemic. Acute Med Surg. (2022) 9:e747. doi: 10.1002/ams2.747, PMID: 35414940 PMC8987347

[14] Lebowitz A Kotani K Matsuyama Y Matsumura M. Using text mining to analyze reflective essays from Japanese medical students after rural community placement. BMC Med Educ. (2020) 20:38–7. doi: 10.1186/s12909-020-1951-x, PMID: 32028939 PMC7006181

[15] Lin CW Lin MJ Wen CC Chu SY. A word-count approach to analyze linguistic patterns in the reflective writings of medical students. Med Educ Online. (2016) 21:29522. doi: 10.3402/meo.v21.29522, PMID: 26838331 PMC4737714

[16] Nakagawa K Asakawa Y Yamada K Ushikubo M Yoshida T Yamaguchi H. Benefits of off-campus education for students in the health sciences: a text-mining analysis. BMC Med Educ. (2012) 12:84. doi: 10.1186/1472-6920-12-84, PMID: 22928985 PMC3479041

[17] Ng HKY. How do students’ learning goals differ? A text mining approach to reveal the individual differences. Front Educ. (2024) 8:1265193. doi: 10.3389/feduc.2023.1265193

[18] Zimmerman BJ Kitsantas A. Comparing students’ self-discipline and self-regulation measures and their prediction of academic achievement. Contemp Educ Psychol. (2014) 39:145–55. doi: 10.1016/j.cedpsych.2014.03.004

[19] Fryer LK Ginns P Walker R. Between students' instrumental goals and how they learn: goal content is the gap to mind. Br J Educ Psychol. (2014) 84:612–30. doi: 10.1111/bjep.12052 25160115

[20] Torok HM Torre D Elnicki DM. Themes and characteristics of medical students’ self-identified clerkship learning goals: a quasi-statistical qualitative study. Acad Med. (2009) 84:S58–62. doi: 10.1097/ACM.0b013e3181b38c71, PMID: 19907388

[21] Jiang L Elen J. Why do learning goals (not) work: a reexamination of the hypothesized effectiveness of learning goals based on students’ behaviour and cognitive processes. Educ Technol Res Dev. (2011) 59:553–73. doi: 10.1007/s11423-011-9200-y

[22] Cheung E. Goal setting as motivational tool in student's self-regulated learning. Educ Res Q. (2004) 27:3–9.

[23] Kuwaki K. [Assessmentor, a system for visualization of learning outcomes to link to teaching and learning management] Kyougaku management ni tsunageru tame no gakushu seika system “Assessmentor” (in Japanese). Univ Coll Manag. (2021) 17:26–34.

[24] Higuchi K. A two-step approach to quantitative content analysis: KH coder tutorial using Anne of green gables (part I). Ritsumeikan Soc Sci Rev. (2016) 52:77–91.

[25] Higuchi K. A two-step approach to quantitative content analysis: KH coder tutorial using Anne of green gables (part II). Ritsumeikan Soc Sci Rev. (2017) 52:137–47.

[26] Ward JH. Hierarchical grouping to optimize an objective function. J Am Stat Assoc. (1963) 58:236–44. doi: 10.1080/01621459.1963.10500845

[27] Palmer S. Crowdsourcing customer needs for product design using text analytics In: Proceedings of the world congress on engineering. London: (2016)

[28] Dunham L Dekhtyar M Gruener G CichoskiKelly E Deitz J Elliott D. Medical student perceptions of the learning environment in medical school change as students transition to clinical training in undergraduate medical school. Teach Learn Med. (2017) 29:383–91. doi: 10.1080/10401334.2017.1297712, PMID: 28318319

[29] Nygren-Bonnier M Högstedt K Laurell A Boström C. First and final year physiotherapy students’ expectations of their future profession. Physiother Theory Pract. (2022) 39:2366–76. doi: 10.1080/09593985.2022.2075295, PMID: 35583494

[30] Beltrán-Velasco AI Ruisoto-Palomera P Bellido-Esteban A García-Mateos M Clemente-Suárez VJ. Analysis of psychophysiological stress response in higher education students undergoing clinical practice evaluation. J Med Syst. (2019) 43:68–7. doi: 10.1007/s10916-019-1187-7, PMID: 30734084

[31] Brooke T Brown M Orr R Gough S. Stress and burnout: exploring postgraduate physiotherapy students’ experiences and coping strategies. BMC Med Educ. (2020) 20:433–11. doi: 10.1186/s12909-020-02360-6, PMID: 33198724 PMC7670805

[32] Gallasch D Conlon-Leard A Hardy M Phillips A Van Kessel G Stiller K. Variable levels of stress and anxiety reported by physiotherapy students during clinical placements: a cohort study. Physiotherapy. (2022) 114:38–46. doi: 10.1016/j.physio.2021.12.002, PMID: 35091327

[33] Judd BK Alison JA Waters D Gordon CJ. Comparison of psychophysiological stress in physiotherapy students undertaking simulation and hospital-based clinical education. Simul Healthc. (2016) 11:271–7. doi: 10.1097/sih.0000000000000155, PMID: 27093508

[34] Rezaee M Rassafiani M Khankeh H Hosseini MA. Experiences of occupational therapy students in the first fieldwork education: a qualitative study. Med J Islam Repub Iran. (2014) 28:110–5. PMID: 25664311 PMC4301239

[35] Patton MQ. Enhancing the quality and credibility of qualitative analysis. Health Serv Res. (1999) 34:1189–208. PMID: 10591279 PMC1089059

